# Spatially explicit analysis of field inventories for national forest carbon monitoring

**DOI:** 10.1186/s13021-016-0050-0

**Published:** 2016-06-07

**Authors:** David C. Marvin, Gregory P. Asner

**Affiliations:** Department of Global Ecology, Carnegie Institution for Science, 260 Panama St., Stanford, CA 94305 USA

**Keywords:** Field sampling, Forest carbon stocks, Tropical forest, Carnegie Airborne Observatory

## Abstract

**Background:**

Tropical forests provide a crucial carbon sink for a sizable portion of annual global CO_2_ emissions. Policies that incentivize tropical forest conservation by monetizing forest carbon ultimately depend on accurate estimates of national carbon stocks, which are often based on field inventory sampling. As an exercise to understand the limitations of field inventory sampling, we tested whether two common field-plot sampling approaches could accurately estimate carbon stocks across approximately 76 million ha of Perúvian forests. A 1-ha resolution LiDAR-based map of carbon stocks was used as a model of the country’s carbon geography.

**Results:**

Both field inventory sampling approaches worked well in estimating total national carbon stocks, almost always falling within 10 % of the model national total. However, the sampling approaches were unable to produce accurate spatially-explicit estimates of the carbon geography of Perú, with estimates falling within 10 % of the model carbon geography across no more than 44 % of the country. We did not find any associations between carbon stock errors from the field plot estimates and six different environmental variables.

**Conclusions:**

Field inventory plot sampling does not provide accurate carbon geography for a tropical country with wide ranging environmental gradients such as Perú. The lack of association between estimated carbon errors and environmental variables suggests field inventory sampling results from other nations would not differ from those reported here. Tropical forest nations should understand the risks associated with primarily field-based sampling approaches, and consider alternatives leading to more effective forest conservation and climate change mitigation.

**Electronic supplementary material:**

The online version of this article (doi:10.1186/s13021-016-0050-0) contains supplementary material, which is available to authorized users.

## Background

More atmospheric carbon is absorbed and stored by tropical forests than any other terrestrial ecosystem on Earth [1]. This crucial ecosystem service provides a carbon sink larger than what is emitted by fossil fuel combustion across the entire European Union each year [2, 3]. Policies that monetize the amount of carbon stored annually by a hectare of tropical forest seek to incentivize forest conservation by making it more economical to leave the forest intact than to degrade or deforest the land. The resulting economic boon for landowners and countries that reduce deforestation and degradation also results in increased carbon sequestration in the form of woody biomass, reducing global net carbon emissions. For such policies to be successful, the uncertainty in standing carbon stocks and change (flux) must be reduced.

Accurate carbon flux calculations necessitate accurate estimates of standing carbon stocks at one or more time periods, unless carbon fluxes are measured using more direct methods (e.g., eddy covariance, atmospheric inversion). Both the price of carbon and the efficacy of climate change mitigation can be negatively affected by uncertainty in our understanding of carbon stocks, requiring the deployment of methods to make highly accurate spatial and temporal estimates of forest carbon. As forest carbon stock uncertainties increase, the monetary value of that carbon is decreased through a sliding scale discount [4, 5], reducing investment opportunities and the economic benefits accrued by countries and landowners. Depending on the baseline levels of deforestation and carbon storage rates, Kohl et al. [4] found in a simulation study that many countries would generate no economic benefit with total errors in carbon estimates exceeding 5 % unless baseline deforestation rates are very high. Ultimately the power of these policies to increase forest conservation and mitigate climate change may rely on our ability to accurately quantify forest carbon in a spatially explicit manner, as opposed to generalized estimates for total carbon stocks of a landscape, habitat type, or eco-region.

Spatially explicit maps of forest carbon, or carbon geographies, allow for multi-stakeholder engagement at subnational levels [6]. Landholders and agencies within tropical countries control forest assets across a variety of spatial extents. Accordingly, knowledge of carbon stocks is needed at scales commensurate with the activities of these subnational stakeholders. Providing generalized carbon stock estimates for a given area, rather than a carbon geography, removes important spatial heterogeneity in forest carbon that could lead to over- or underestimates of carbon stocks, and consequently, the potential for poor land-use decisions. Both field inventories [7] and forest fragmentation [8] may introduce bias into forest carbon stock estimates; a bias that arises when local landscape heterogeneity is disregarded.

Forest carbon inventories typically rely (or are encouraged to rely) on a network of field plots installed either on a regular systematic grid or a random stratified grid [9–11]. Importantly, the IPCC guidelines for estimating and measuring carbon direct countries to use these sampling approaches [12, 13]. Although these guidelines are primarily designed for total national or average carbon density estimates, they are sometimes used for carbon geographies as well [14, 15]. Field plots are usually 1-ha in size (the generally accepted standard for a forest plot inventory [16]) but forest inventories that use smaller plot designs run the risk of the plots being even less representative of the surrounding forest [17]. The data collected in the field can then be combined with remote sensing data in two general ways: stratify-and-multiply, and model-linked. The stratify-and-multiply approach [18] uses remote sensing data to partition a country by land cover, climate, or other environmental (or biogeochemical) strata, or uses a regular (systematic) grid in place of partitioning by strata. Each unique stratum (or grid cell for systematic sampling) is assigned the field plot-estimated carbon stock and multiplied by the total area within the stratum. Alternatively in a model-linked approach, a model is calibrated to link field plot-estimated carbon stocks with multiple environmental variables at the location of the plots (or an intermediate remote sensing product such as LiDAR tree height), and the model applied to the entirety of the dataset for which there is no field data (or intermediate data product). The integration of remote sensing data with field data is preferable, but countries may not have the capacity to work with remote sensing data, due to a lack of either in-country expertise, funding to outsource the work, access to technology, or sufficient remotely sensed data [e.g., [19], [20]. In cases where field plot sampling cannot be supplemented by sufficient remote sensing data and/or modeling, spatial heterogeneity in forest carbon is necessarily disregarded because of the time and expense required for the massive field sampling needed to sufficiently capture such heterogeneity [7].

**Fig. 1:**
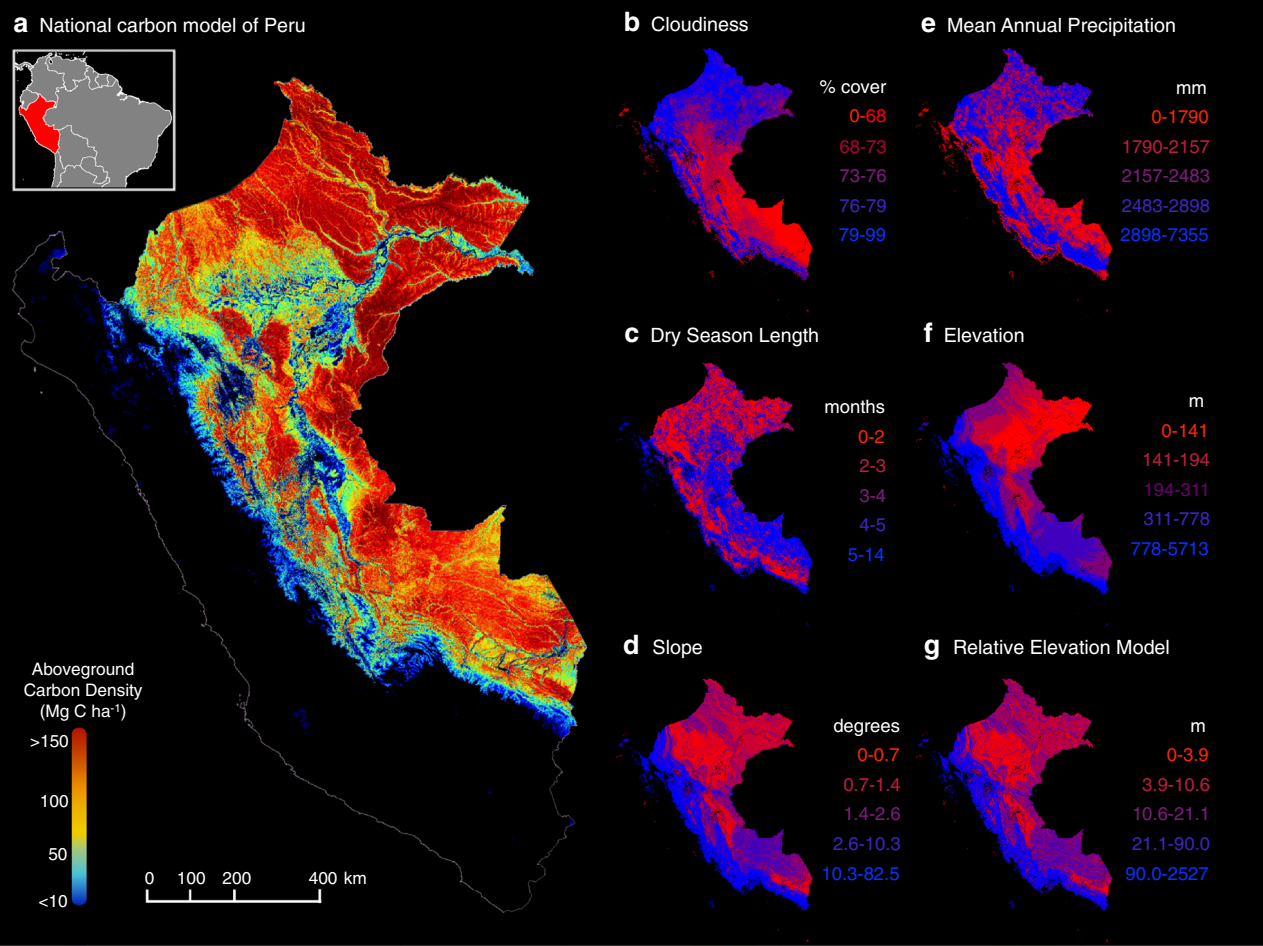
Maps used for the analyses. **a** The model aboveground carbon density (ACD) map of Perú at 1-ha resolution with all non-forested areas masked out (adapted from [6]), and the strata binned and* colored* by quintiles for **b** cloudiness, **c** dry season length, **d** slope, **e** mean annual precipitation, **f** elevation and **g** relative elevation (see “Methods” section)

While field plot sampling alone is commonly used to scale ecosystem properties and processes from local-to-regional scales, the efficacy of this approach has never been assessed at the appropriate scale. As a result, we do not know whether field carbon inventories can be used to create accurate spatially-explicit maps of national carbon geography at one-ha resolution. Here, using Perú as an example country, we examine both systematic and stratified random field-based sampling designs with a LiDAR-estimated national carbon map spanning all 76 million ha of intact and recovering forest land (Fig. 1a) as a model of the country’s true carbon stocks (see ‘‘Methods’’ section for more detail). Hereafter, we refer to this LiDAR-estimated carbon map as “the model carbon stock” or “the model aboveground carbon density (ACD).” For both sampling designs we examine the stratify-and-multiply approach (i.e., applying each sampled value to its corresponding unsampled population at the original spatial resolution of the map) and a model-linked approach using the random forest machine learning algorithm to create spatially explicit carbon geographies of the country. We assume perfect a priori knowledge of the country’s stratification, and assume that each forest plot location is accessible. This is a theoretical exercise using no field inventory data; rather all values are extracted from the carbon map as if it reflected reality. We chose a 10 % interval (i.e., ±5 %) around the true carbon stock value as our threshold for an estimate to be considered accurate. We asked the following questions. (i) What sampling intensity is needed to accurately estimate the total national carbon stock? (ii) Can either sampling method accurately estimate the national carbon geography at 1-ha resolution? (iii) What topographic and climatic variables cause increased error in carbon stock estimates?

## Results and discussion

### Total national carbon stocks

#### Systematic grid

**Fig. 2:**
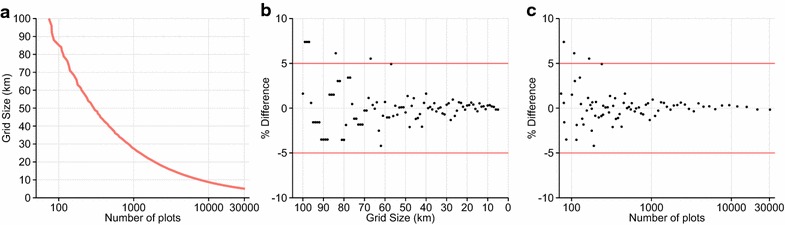
National total carbon stock estimation using systematic grid sampling showing **a** the relationship between the sampling grid dimensions and number of 1-ha field plots, **b** the percent difference between total field plot estimated and total model national carbon stocks by grid size and **c** same as in **b** but with the number of plots required by the grid sampling on the* x-axis* (*log scale*)

A systematic sampling grid with dimensions of ≤56 km (requiring a minimum of 236 field plots) resulted in <2.5 % difference between the total field plot-estimated and the total model national forest carbon stock (Fig. 2). Even for systematic grids of larger spatial grains, estimates tended to stay within the 10 % threshold, with just a few of the large grid sizes falling outside that range. This demonstrates that it is possible to estimate national total forest carbon stock quite accurately with minimal sampling effort using a systematic grid.

#### Stratified random

Using a stratified random sampling approach, we find that only a single plot per substratum is needed to estimate the total carbon stock of Perú to within 10 % of the total model national carbon stock. A maximum of 5398 plots nationwide are needed to sample all combinations of strata representing more than 1000 ha. In fact, only the largest 15 % of the substrata (by area) need to be sampled with a single field plot to yield an accurate estimate of the total national carbon stock, requiring a minimum of 810 plots (Additional file 1: Fig. S1).

Both sampling methods result in accurate estimates of Perú’s total carbon stock, usually falling within a few percent of the model total. However, the systematic grid approach requires less sampling intensity compared to the stratified random, needing only single plots spaced as far apart as 56 km over the entire country. Systematic grid sampling is the cheapest and most efficient way to accurately sample total national carbon stocks of even a country with highly heterogeneous forest carbon distributions.

While accurate estimates of total carbon at the national and regional scale are important for understanding broad geographic patterns of carbon distributions, they are very difficult to use in applications of carbon conservation and monetization, activities that are highly spatially dependent and vary at local, landscape, and sub-regional scales. Improved knowledge of spatial variation in carbon stocks, ideally at the hectare resolution, will enhance policies that seek to incentivize conservation and climate change mitigation through carbon valuation. Maps of national local carbon geographies must be developed with particular attention to accuracy, repeatability, and cost.

### Carbon geography

#### Systematic grid

**Fig. 3:**
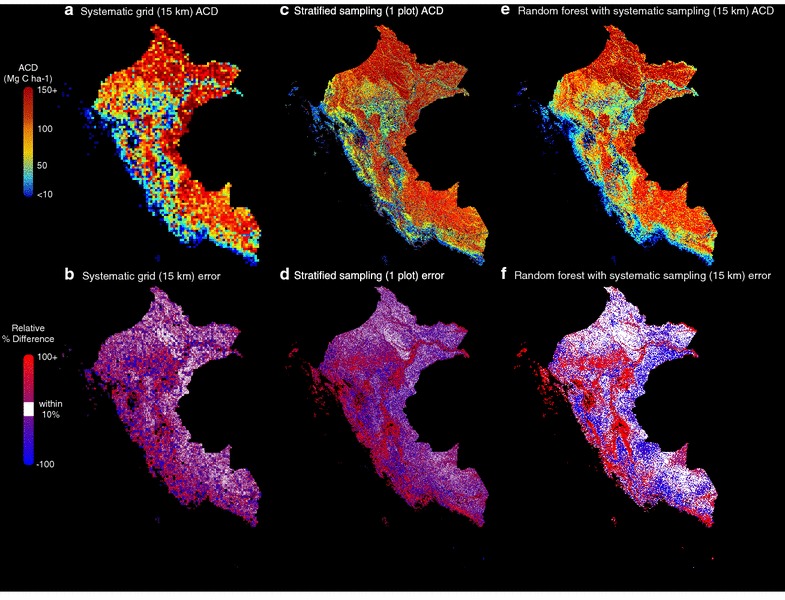
Results of the sampling and upscaling. **a** Estimated ACD using a 15 km systematic grid sampling approach, **b** percent relative difference between model ACD (from Fig. 1a) and **a**, **c** ACD using a 1-plot stratified sampling approach, **d** percent relative difference between model ACD and **c**, **e** ACD using random forest upscaling trained with 15 km systematic sampling grid and **f** percent relative difference between model ACD and (**e**)

**Fig. 4:**
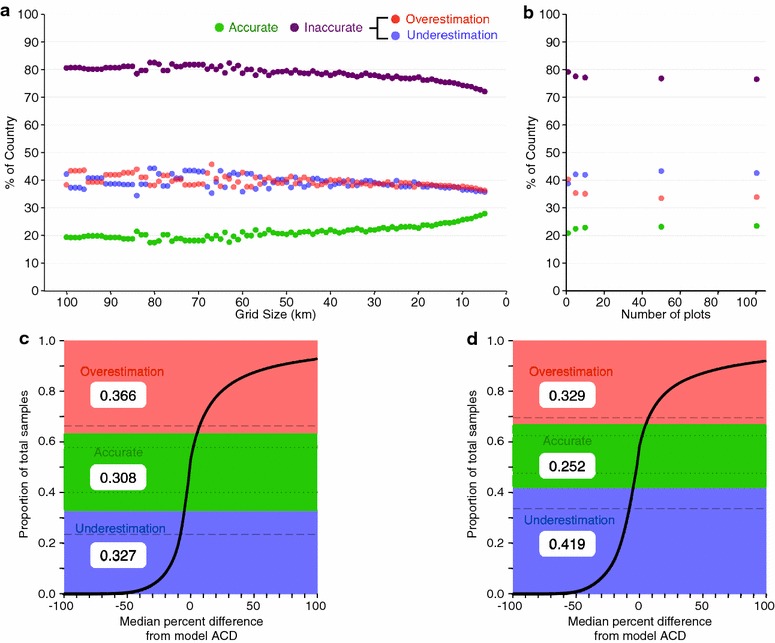
Accuracy assessment of plot sampling when using stratify-and-multiply upscaling to within at least 10 % of the model ACD using **a** systematic grid sampling and **b** stratified random sampling with same legend as in (**a**). The empirical cumulative distribution of the percent relative difference between the model ACD and the median estimated ACD error across **c** all systematic sampling grids and **d** all stratified random sampling plot sets (see ‘‘Methods’’ section). The* coloring* in **c**, **d** show the proportions that overestimate (*red*), underestimate (*blue*), and correctly estimate (*green*) within 10 % of the model ACD value. *Horizontal broken lines* in **c**, **d** show how the proportion correctly estimated would change with a more stringent (5 %, *dotted lines*) or less stringent (15 %, *dashed line*) error threshold

**Fig. 5:**
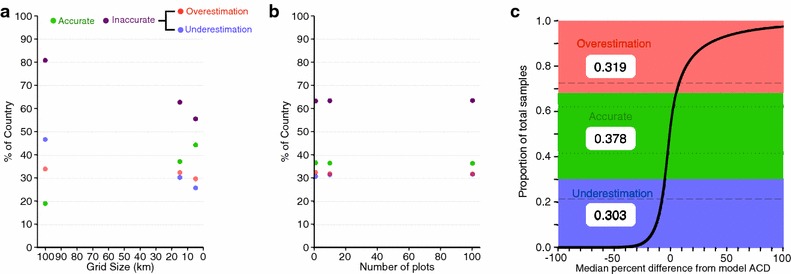
Accuracy assessment of plot sampling when using model-linked (random forest) upscaling to within at least 10 % of the model ACD using **a** systematic grid sampling and **b** stratified random sampling with same legend as in (**a**). The empirical cumulative distribution of the percent relative difference between the model ACD and the median estimated ACD error across **c** all systematic sampling grids and all stratified random sampling plot sets (see ‘‘Methods’’ section). The* coloring* in **c** show the proportions that overestimate (*red*), underestimate (*blue*), and correctly estimate (*green*) within 10 % of the model ACD value, and *horizontal broken lines* show how the proportion correctly estimated would change with a more stringent (5 %, *dotted line*s) or less stringent (15 %, *dashed line*) error threshold

Using a systematic sampling grid to estimate ACD and then upscaling the result at 1-ha resolution using the stratify-and-multiply approach leads to a large percentage of inaccurate estimates across the country (i.e., field plot-estimated ACD failed to be within 10 % of the model ACD) (Fig. 3a, c). Regardless of the sampling grid size, this approach produces inaccurate estimates across approximately 78 % of the country (Fig. 4a, c). The inaccurate estimates are quite consistent, 82 % using a 100 km grid (requiring just 74 1-ha plots) vs. 71 % using a 5 km grid (requiring >30,000 1-ha plots), suggesting that this method performs poorly in creating an accurate map of Perú’s carbon geography. Using a model-linked random forest approach, rather than stratify-and-multiply upscaling, improves the carbon map of Perú but still results in inaccurate estimates across more than half of the country (Fig. 5a, c). Using a systematic grid of 15 km (requiring approximately 3400 1-ha plots) as the training dataset for a random forest model, 63 % of the country is still inaccurately estimated. Even using the 5 km grid results in 56 % of the country inaccurately estimated using random forest upscaling, but still requires a field plot sampling intensity of 30,459 1-ha plots to produce the training dataset (Figs. 3e, f, 5a).

#### Stratified random

The stratified random sampling stratify-and-multiply approach did not perform much better than the systematic grid stratify-and-multiply approach in estimating the carbon geography of Perú. The distribution of inaccurate estimates was similar to the systematic grid approach (Fig. 3b, d), and resulted in nearly the same amount of total inaccurate estimates across the country (Fig. 4b, d). Using only a single randomly placed plot per substrata (requiring around 5400 plots) leads to 79 % of the country inaccurately estimated. There is barely any improvement—with 76 % of the country inaccurately estimated—when the number of plots randomly placed inside a substrata is increased to 100 (requiring around 540,000 plots). Again the model-linked random forest upscaling approach did not substantially increase the area accurately mapped (Fig. 5b, c), with 63 % of the country inaccurately estimated regardless of the number of plots used per substratum (1, 5 or 100 plots).

Neither field plot sampling approach assessed here was able to accurately estimate the carbon geography at the 1-ha scale across more than 25–44 % of Perú. While field plot systematic sampling tended to equally under- and overestimate the model ACD across the country, the field plot stratified sampling consistently underestimated model ACD at the 1-ha scale (Fig. 4b, d). Chronically underestimating local carbon stocks will artificially deflate the per-hectare value of carbon, leading to reduced conservation incentives by landholders. Even when the total sampling is increased to an impractical number of plots (i.e., tens- to hundreds-of-thousands), the accuracy does not improve substantially. This demonstrates that neither sampling approach would be an appropriate choice for developing national maps of carbon geography, or changes in carbon geography via emissions or sequestration.

### Underlying drivers of uncertainty

**Fig. 6:**
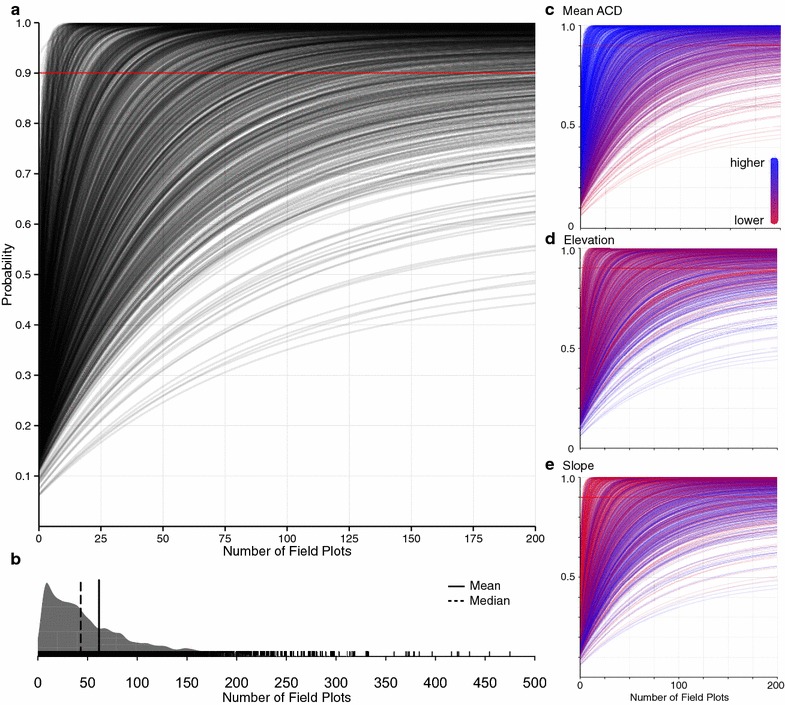
Results of the uncertainty simulations. **a** Number of field plots needed to reliably (pr=0.9, *red line*) estimate the mean ACD (Mg C ha^−1^) of a substratum using quintile binning (note: a random selection of 1000 substrata are *plotted for clarity*). **b** Frequency distribution of the number of field plots needed to reliably estimate a substratum’s mean ACD. Same as in **a** but* colored* by the substratum’s **c** mean ACD, **d** elevation and **e** slope. Further topographic and climatic variables are shown in Additional file 1: Fig. S3

To understand whether there are particular strata or carbon values that lead to higher field plot estimated ACD errors, we used simulations to examine the number of field plots needed to reliably estimate (probability ≥ 0.9) the mean ACD of all substrata (unique combinations of the 6 quintile-binned strata) (Fig. 1b–g) to within 10 % of the model ACD (Fig. 6a, b). While most substrata (81 %) can be accurately estimated at a certain sampling density, some never reach the 0.9 probability threshold at any sampling density, meaning they are so heterogeneous that field plot sampling is not feasible. The median number of field plots needed to reliably sample the mean model ACD of any substratum is 43 (mean = 61; Fig. 6b).

Further examination of the simulation outcomes by strata or carbon value shows a few strong patterns. Substrata that had higher mean model ACD values require fewer randomly placed plots to accurately sample the mean model ACD (Fig. 6c). This implies that the heteroskedasticity in the relationship used to develop the model ACD map (see ‘‘Methods’’ section) is not a major factor in the inability of the field plot sampling strategies to reproduce the model ACD. The strata with lower elevation and slope values require fewer plots to accurately sample the mean ACD (Fig. 6d, e), indicating that these areas are more likely to have lower heterogeneity in their carbon stocks. The other strata showed weaker or no patterns (Additional file 1: Fig. S2). Similar patterns were found when the same simulations were conducted with strata that were binned by equal range (Additional file 1: Figs. S3–S5).

**Fig. 7:**
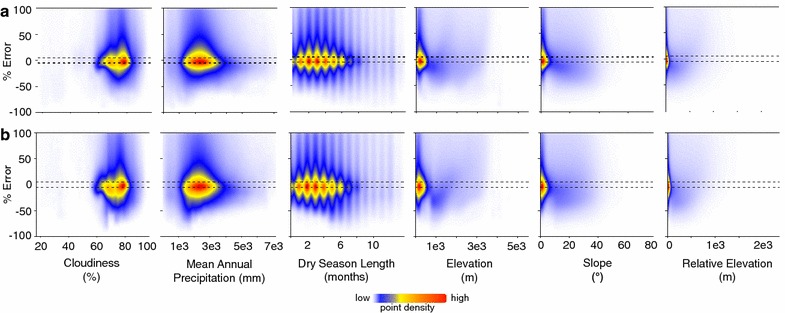
Underlying drivers of uncertainty. The percent relative difference between the model ACD and the median estimated ACD error across **a** all systematic sampling grids and **b** all stratified random sampling plot sets (see ‘‘Methods’’ section), plotted against each non-binned stratum at the 1-ha scale.* Plots* are composed of a randomly selected 10 % of the total dataset (approximately 7.6 million hectares), with the point density* color scale* on a square root transformation for plotting clarity. *Horizontal dashed black lines* show the 10 % accuracy range

We also extracted the relative percent error between the field plot sampling estimated ACD and the model ACD, and graphed this against each stratum on a hectare-by-hectare basis (Fig. 7). Both field-plot sampling methods produced similar patterns in their estimation errors with each stratum. None of the strata show a particularly clear overall association with their ACD estimation error. Instead, generally the spread in error densities seem either restricted to low strata values in the case of elevation, slope, and relative elevation, or are more widespread across the strata values in the case of cloudiness, mean annual precipitation, and dry season length. This is probably more a reflection of the underlying data distributions than patterns in the relationship between estimation error and environmental variables (i.e., where there are more data you would expect a larger spread in the errors).

The lack of strong and clear trends between the percent relative error and the strata used in this analysis further reduces the utility of plot sampling for creating accurate, spatially explicit national carbon maps. If there were particular strata or subsets of strata that were underlying the resulting errors, then these could be isolated and sampled differently from the remaining areas of the country. The large errors within and between the strata indicate that widespread ACD estimation errors will be unavoidable when using a field-plot sampling strategy.

## Conclusion

Creating a national carbon geography using field inventory plot sampling is unlikely to produce accurate results that can be deployed for use in spatially explicit actions to reduce carbon emissions. In this exercise we find that two common methods for large-scale field sampling fail to produce accurate (within a 10 % interval around the model ACD) carbon estimates for any more than 44 % of the total forested areas of Perú (approximately 76 million ha), based on a model of carbon geography. This holds true even when 12 layers of remote sensing imagery were used to upscale 30,459 field plot samples using a machine learning algorithm. While both sampling strategies can produce accurate estimates of the *total* carbon content of Perú using relatively few plots, the local carbon geographies of countries are far more important in a carbon valuation and carbon sequestration context. Without accurate, one-hectare resolution carbon geographies, land use decisions by subnational stakeholders (individual landowners and agencies) may be based on insufficient or biased information.

Perú is an ideal tropical country for this type of exercise because it hosts a wide range of topographic, biotic, geologic, and climatic variation resulting in highly heterogeneous landscape carbon distributions. While some countries may have very low carbon heterogeneity, most efforts to map national carbon stocks will face the issue of high sampling errors resulting from non-homogenous carbon distributions. This means our results are likely applicable to most tropical countries, or at least to substantial portions of any particular country.

Field inventories could be targeted toward more intensive sampling of areas likely to have higher carbon heterogeneity, potentially reducing the estimation uncertainties of any local carbon geography. This is the basic premise underlying any stratification approach [11], whether the basic stratification used here or those that incorporate remote sensing data. However, we did not find any variables driving the errors in local carbon estimates among the six topographic and climatic strata tested. These six strata are the environmental variables that best explain total variation in carbon across Perú [6]. Therefore, countries using field-based carbon mapping will find it difficult a priori to target sampling toward areas of potential high carbon estimation uncertainty. Moreover, even if a country were to have perfect knowledge of its strata, as in the stratification exercise presented here, field plot sampling still cannot reproduce the model ACD without incurring substantial errors across much of the country.

Countries seeking to value their forest carbon reserves for conservation and climate change mitigation must look beyond the sole use of field plot sampling. Of course, field inventory plots are critical for understanding local-scale ecological processes and for calibration/validation of remote sensing data. (We emphasize here the important distinction between the use of field plots as calibration/validation for remote sensing products, and using remote sensing products to scale field plot results to larger areas). While field plots are integral to remote sensing campaigns, they are not designed for, and do not perform well at, producing spatially explicit estimates of forest carbon stocks [21]—even when using satellite based sensors for upscaling. Field inventories are hard pressed to adequately capture the link between remotely sensed environmental variables and estimated carbon stocks. For example, 30,000 field plots spread across Perú—an extremely ambitious sampling effort for most countries—is still only 0.5 % of the total area (6.76 million ha) sampled by airborne LiDAR and used to create the model ACD map [6].

Instead tropical countries should look toward airborne and spaceborne sensors to fulfill the need for improved wall-to-wall aboveground carbon maps at hectare scales. The LiDAR sensor on the future ICESat-2 satellite is unlikely to accurately measure tropical forest carbon [22] and the GEDI sensor will be a short-term (1–2 year) mission on the International Space Station [23]. The lack of a long-term spaceborne sensor to measure global forest carbon suggests that we must rely on airborne platforms to carry out the bulk of national carbon sampling for at least the next decade. Airborne LiDAR can now successfully map forest carbon stocks at high-resolution at subnational to national scales at high accuracy and extremely low cost on a per-hectare basis if operated non-commercially; hundreds of thousands of hectares can be mapped per day at accuracies that approach or match field plot based sampling [24, 25]. The economies of scale achieved by a single airborne LiDAR sensor leads to far more cost-effective and accurate carbon mapping that is easily repeatable—yielding estimates of carbon net changes over time—gives this method of mapping a drastic advantage over field plot sampling.

No doubt tropical forests and their substantial carbon sink capacities will play a major role during the implementation of agreements forged during the December 2015 UNFCCC climate conference in Paris. Tropical forest nations must be ready to respond with accurate assessments of their carbon stocks and reliably monitor their changes over time. While field inventory plots have long been the standard for meeting these challenges, with tens of millions of dollars spent on plot implementation and infrastructure, this should not preclude a move toward more accurate and cost-effective forest carbon mapping.

## Methods

### Study area

Perú is an ideal test case for investigating whether field plot sampling can accurately map carbon because its forests span a wide range of topographic, climatic, floristic, and geologic variables. Heterogeneity in aboveground carbon density (ACD) is driven by combinations of these variables, and higher ACD heterogeneity leads to less accurate field plot estimates of ACD [7]. Results from within the diverse environmental gradients of Perú can then be applied to other countries of similar conditions.

We used the 1-ha resolution aboveground carbon density map of Perú created by Asner et al. [6] as a model of the “true” carbon stocks of the country. We refer to this LiDAR-estimated carbon map throughout the paper as “the model carbon stock” or “the model aboveground carbon density (ACD).” The model ACD map was produced from a countrywide airborne LiDAR campaign with the Carnegie Airborne Observatory (CAO; cao.carnegiescience.edu). The LiDAR data were integrated with high-resolution satellite imaging, a large field plot sampling network (to calibrate/validate the LiDAR carbon mapping), and an advanced geospatial scaling algorithm [for more details see 6]. Please refer to Fig. S5 of [6] for model validation results, which compared 536,874 ha of LiDAR-measured forest TCH that were not used to train the model-estimated TCH of those same locations. The R^2^ is 0.78 and the RMSE is 3.50 m. While the underlying relationship between LiDAR measured-TCH and estimated ACD may be heteroskedastic, with a higher ACD errors found at taller TCH, the relative ACD uncertainty that results is low (i.e., <20 % for high ACD (>120 MgC ha^−1^) lowland forests).

We masked this map to exclude areas that are not forested, using a mean per hectare threshold of >70 % photosynthetic vegetation and >2 m top-of-canopy height (TCH). The photosynthetic vegetation map was created from a national-scale mapping of Perú using the CLASlite algorithm with mosaicked Landsat satellite imagery [26]. The TCH map is the underlying dataset used to produce the ACD map of Perú [6]. This left a forested area of 76,457,286 ha for the analysis. While this is 59.5 % of Perú by land area, it contains 98.5 % of the total carbon of the entire country (6.798 Pg). All analyses were performed using R statistical software [27] with geospatial manipulations and analyses performed using the ‘raster’ package [28].

### Environmental stratification layers

We compiled a set of six co-aligned, 1-ha resolution environmental stratification layers (hereafter ‘strata’) comprised of topographic and climatic variables covering all of Perú. Cloudiness (%) was created from long-term (2000–2010) NASA moderate resolution imaging spectroradiometer (MODIS) data and described in further detail by [6]. Mean annual precipitation (MAP) was calculated from NASA Tropical Rainfall Measuring Mission (TRMM) 2b31 monthly data (1998–2011), and dry season length (DSL) was calculated using the same TRMM data for all months with less than 100 mm of precipitation. Elevation, slope, and a relative elevation model (REM) were created from NASA Shuttle Radar Topography Mission (SRTM) at 90 m resolution. Relative elevation is a proxy for vegetation related water resources, and is developed by calculating the height of the ground above nearest water body [29]. Each stratum was masked by the same forest mask described above.

### Field plot sampling designs

We tested two commonly used field plot sampling strategies: systematic sampling and stratified random sampling. This study is not meant to be an exhaustive review of spatial sampling techniques, rather to evaluate two methods that are commonly employed or recommended to map forest carbon over large geographic areas [9, 10, 12, 13]. Therefore, we did not consider other possible spatial sampling designs (e.g., simple random sampling, cluster sampling).

Systematic sampling uses sampling units (i.e., field inventory plots) placed at regular intervals according to an ordering scheme. For the country of Perú, we chose regularly spaced square grids with dimensions ranging from 5–100 km, increasing at 1-km intervals. Only those sampling units on the grid that fall within the bounds of the forest mask were retained (hereafter referred to as systematic grid sampling).

Stratified sampling places sampling units across a region according to pre-defined subregions that are more homogenous than the region as a whole, thereby reducing inherent sampling errors. The degree to which a stratified sampling technique accurately estimates the true population depends largely upon choosing homogenous subregions from which to conduct the subsampling [30]. We used unique combinations of the six strata described above to create these subregions (hereafter referred to as ‘substrata’). First each stratum was binned by quintile, creating 5 categorical classes for each stratum (Fig. 1b–g). Then a map of all unique combinations of the six quintile-binned strata was produced, and any resulting substrata less than 1000 ha in area was excluded (hereafter referred to as ‘quintile-binned substrata’). This resulted in 5398 unique substrata totaling 98.2 % of the forested area used for the analyses. We repeated the above steps, instead binning each stratum by 5 equal subsets of the total range of values of each stratum (hereafter referred to as ‘range-binned substrata’) (Additional file 1: Fig. S3). Again removing any substrata with less than 1000 ha, resulting in 447 substrata representing 99.9 % of the forested area used for the analyses. Sampling units were then randomly selected from within each substratum according to the simulations described below (hereafter referred to as ‘stratified random sampling’).

### Estimating total national carbon stocks

#### Systematic grid

For each systematic sampling grid, the ACD value extracted from the centroid of each sampling grid cell was used as the ACD value for all of the 1-ha values in that sampling grid cell. The total number of grid cells in a systematic grid represents the number of field plots needed to create the estimated ACD map. We produced 96 different estimated ACD maps, each with a spatial resolution corresponding to the sampling grid dimensions used to create the map (5–100 km in 1 km increments). The ACD was summed across the entire country for each of the different estimated ACD maps, and compared to the total of the model ACD map.

#### Stratified random

We used Monte Carlo simulations to determine the probability of a randomly placed set of field inventory plots within each quintile-binned substratum to accurately estimate the total carbon stocks of Perú. For all of the 5398 substrata, a random sample of ACD values drawn from all ACD values of the substratum was selected and used to estimate the mean ACD of the substratum. The mean ACD of each substratum was multiplied by the total number of hectares in the substratum to get the estimated total ACD of that substratum. All estimates of total ACD from the substrata were then summed to get an estimated total ACD of the country, and compared to the total of the model ACD map. This was repeated 5000 times and the probability of correctly estimating the total ACD of Perú to within 10 % was determined. This simulation was run for sample sizes of 1 through 100 field plots in each substratum. We repeated this same simulation, but at each iteration progressively removing the smallest substrata (by area) in increments of 5 %. We used the mean ACD of the sampled substrata to estimate the total ACD of the substrata that were removed from the simulation. We did not test the range-binned substrata because of its poor performance in estimating the mean ACD of a substratum (see ‘‘Uncertainty simulations and extraction’’ section below).

### Estimating national carbon geographies

#### Systematic grid

Each 1-ha resolution systematic grid estimated ACD map (see above) represents a stratify-and-multiply upscaling approach whereby the sampled value from the systematic grid (which can be thought of as stratification in this context) is applied to the corresponding unsampled population (the rest of the 1-ha values in the systematic grid cell). Each estimated ACD map was co-aligned with the model ACD map of Perú, subtracted from the model ACD map, and the difference divided by the model ACD map to get the relative percent difference on a per hectare basis. We also performed a model-linked upscaling approach using random forest machine learning in addition to the stratify-and-multiply upscaling approach. We used the systematic sampling grid values as the training dataset for each random forest model. For the predictors of the model, we used the same 12 contiguous remote sensing layers that were used to create the model ACD map [see 6]. For each systematic grid, a random forest model was fit with the sampled values and their co-aligned values from the remote sensing layers. Each fitted model was then applied to the entire country using the 12 remote sensing layers to create a wall-to-wall random forest estimated ACD map [for more details on the random forest approach see [31]. This map was then compared to the model ACD map in the same manner as above.

#### Stratified random

We chose a subset {1, 5, 10, 50, 100} of field plot sets used to randomly sample each substratum to create a stratified random estimated ACD map using a stratify-and-multiply approach. For each substratum, a field plot set was randomly drawn from all ACD values of the substratum, and the mean of the set was mapped back onto all hectares of that substratum. This produced a set of maps of estimated ACD for the entire country of Perú at 1-ha resolution based on the stratified random sampling approach. For each map created from one of the five field plot sets, the stratified random estimated ACD map was subtracted from the model ACD map, and the difference divided by the model ACD map to get the relative percent difference. Again, we did not test the range-binned strata for the reason described in the preceding section. We also applied a model-linked upscaling approach using the random forest machine learning algorithm. Here the training datasets were composed of the stratified random sample values and their co-aligned values from the 12 remote sensing layers. Random forest models were fit and applied in the same way as described above.

### Uncertainty simulations and extractions

We ran Monte Carlo simulations to find the number of field plots it would take to accurately estimate the mean ACD of a substratum for the stratified random sampling approach. For each substratum, a random sample of ACD values (representing field plots) was selected from all possible ACD values of the substratum and the mean of the sample was compared to the mean of all ACD values of the substratum. This was repeated 5000 times to find the probability that the selected number of field plots would produce an estimate accurate to within 10 % of the mean of the substratum as estimated by the model ACD map. This simulation was run for sample sizes of 1 through 100 field plots. We then mapped the carbon value (either mean or total) and the environmental strata value of each substratum onto the results of these simulations to examine potential patterns.

We co-aligned the spatially explicit maps of estimated ACD created from both sampling approaches with the original (non-binned) strata. For each sampling approach, we created a median estimated ACD map across all of the sampling grids (systematic sampling, 5–100 km) and plot sets (stratified random, {1, 5, 10, 50, 100} plots). We then extracted the percent relative difference between the median estimated ACD map and the model ACD map. We also extracted the six climatic and topographic values associated with each mapped hectare.

## Additional file

**Additional file 1: Figure S1.** The fraction of stratified random substrata needed to estimate model total national carbon stocks. **Figure S2.** Number of field plots needed to reliably estimate the mean model substratum ACD using stratified sampling. **Figure S3.** Strata binned and colored by five equal ranges. **Figure S4.** Number of field plots needed to reliably estimate the mean model substratum ACD using equal range binning. **Figure S5.** Number of field plots needed to reliably estimate the mean model substratum ACD using equal range binning colored by environmental variables.

## Abbreviations

ACD: aboveground carbon density
CAO: Carnegie Airborne Observatory
IPCC: Intergovernmental Panel on Climate Change
LiDAR: Light Detection and Ranging
MAP: mean annual precipitation
MODIS: Moderate Resolution Imaging Spectroradiometer
REM: relative elevation model
SRTM: Shuttle Radar Topography Mission
TCH: top of canopy height
TRMM: Tropical Rainfall Monitoring Mission
UNFCCC: United Nations Framework Convention on Climate Change
