# Supplementary material for: Spatially explicit analysis of field inventories for national forest carbon monitoring
Source: Carbon Balance Manag. 2016 Jun 7;11:9. doi: 10.1186/s13021-016-0050-0 (PMC4894926; doi:10.1186/s13021-016-0050-0)
Supplement: Supplementary file 1 — Additional file 1: Figure S1. The fraction of stratified random substrata needed to estimate model total national carbon stocks. Figure S2. Number of field plots needed to reliably estimate the mean model substratum ACD using stratified sampling. Figure S3. Strata binned and colored by five equal ranges. Figure S4. Number of field plots needed to reliably estimate the mean model substratum ACD using equal range binning. Figure S5. Number of field plots needed to reliably estimate the mean model substratum ACD using equal range binning colored by environmental variables. [file 13021_2016_50_MOESM1_ESM.pdf]

Supplementary Material for

Spatially explicit analysis of field inventories for national forest carbon monitoring

David C. Marvin & Gregory P. Asner

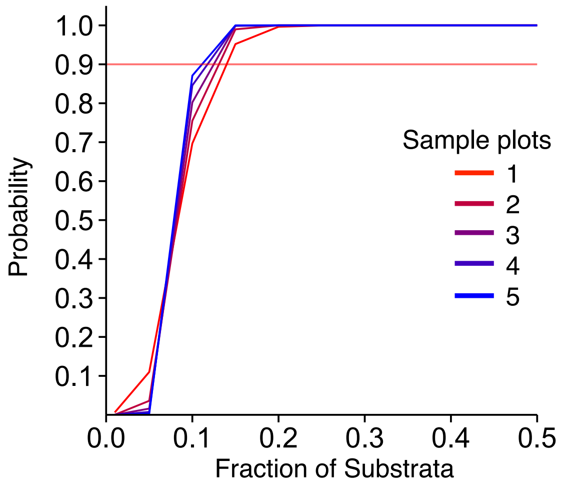

Figure S1. The fraction of stratified random substrata needed to reliably (pr=0.9, red line) estimate the model total national carbon stocks, plotted by the number of sample field plots used per substratum to estimate each substratum's mean ACD.

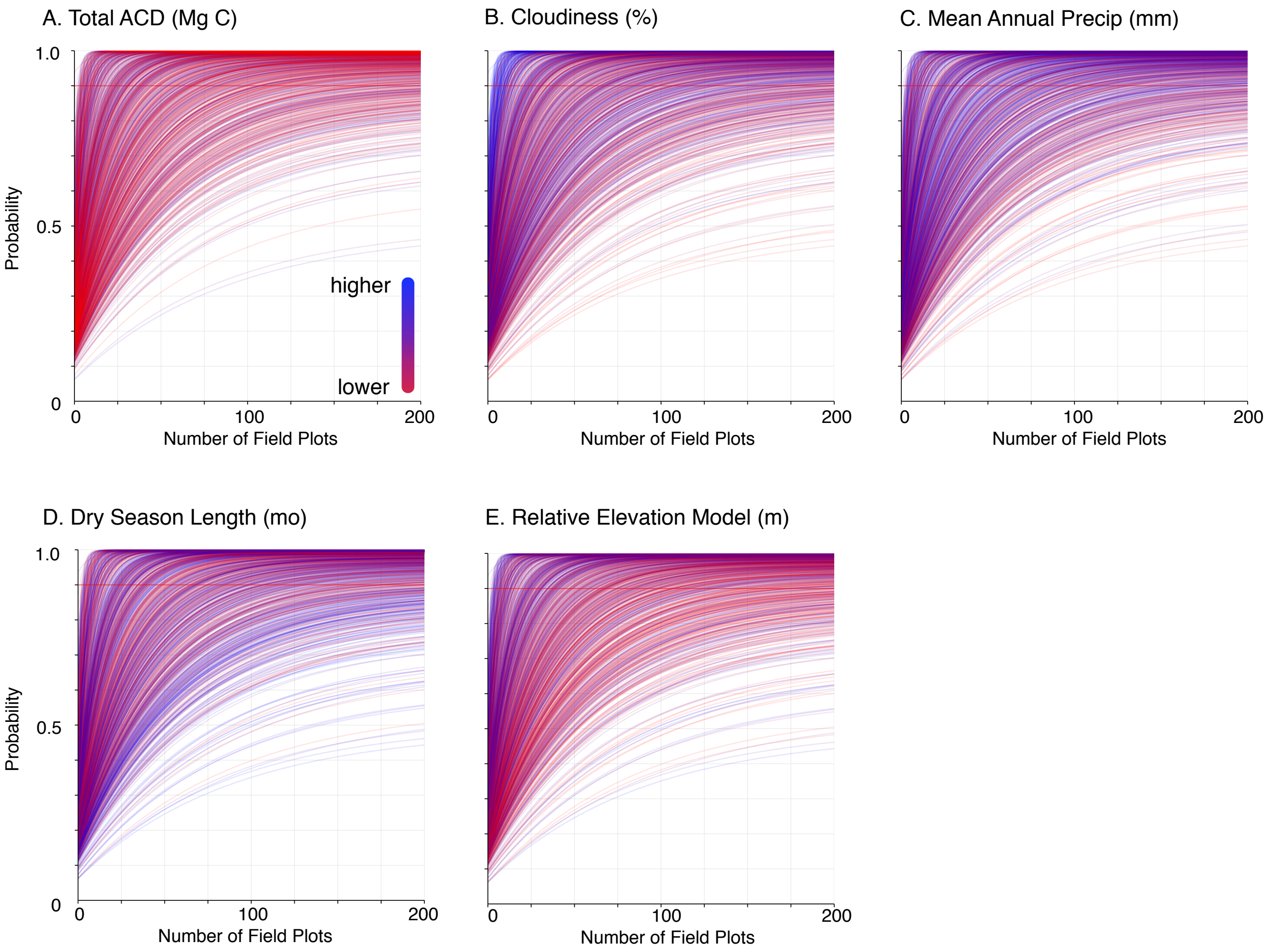

Figure S2. Number of field plots needed to reliably (pr=0.9, red line) estimate the mean model ACD (Mg C ha^{-1}) of a substratum using stratified sampling for (A) total model ACD, (B) cloudiness, (C) mean annual precipitation, (D) dry season length, and (E) relative elevation. Coloring is same as in Fig 1B-D.

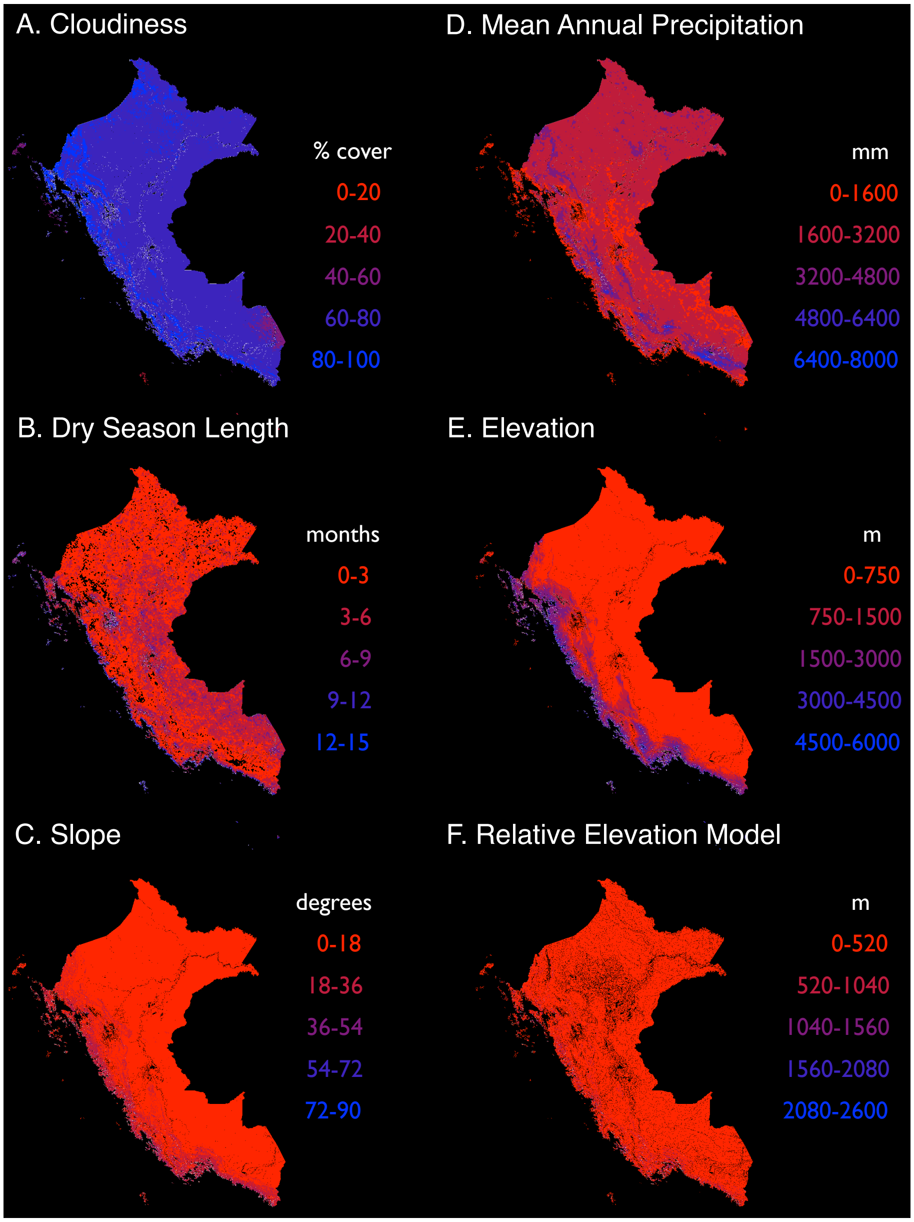

Figure S3. Strata binned and colored by five equal ranges for (A) cloudiness, (B) dry season length, (C) slope, (D) mean annual precipitation, (E) elevation, and (F) relative elevation model (see Methods for description).

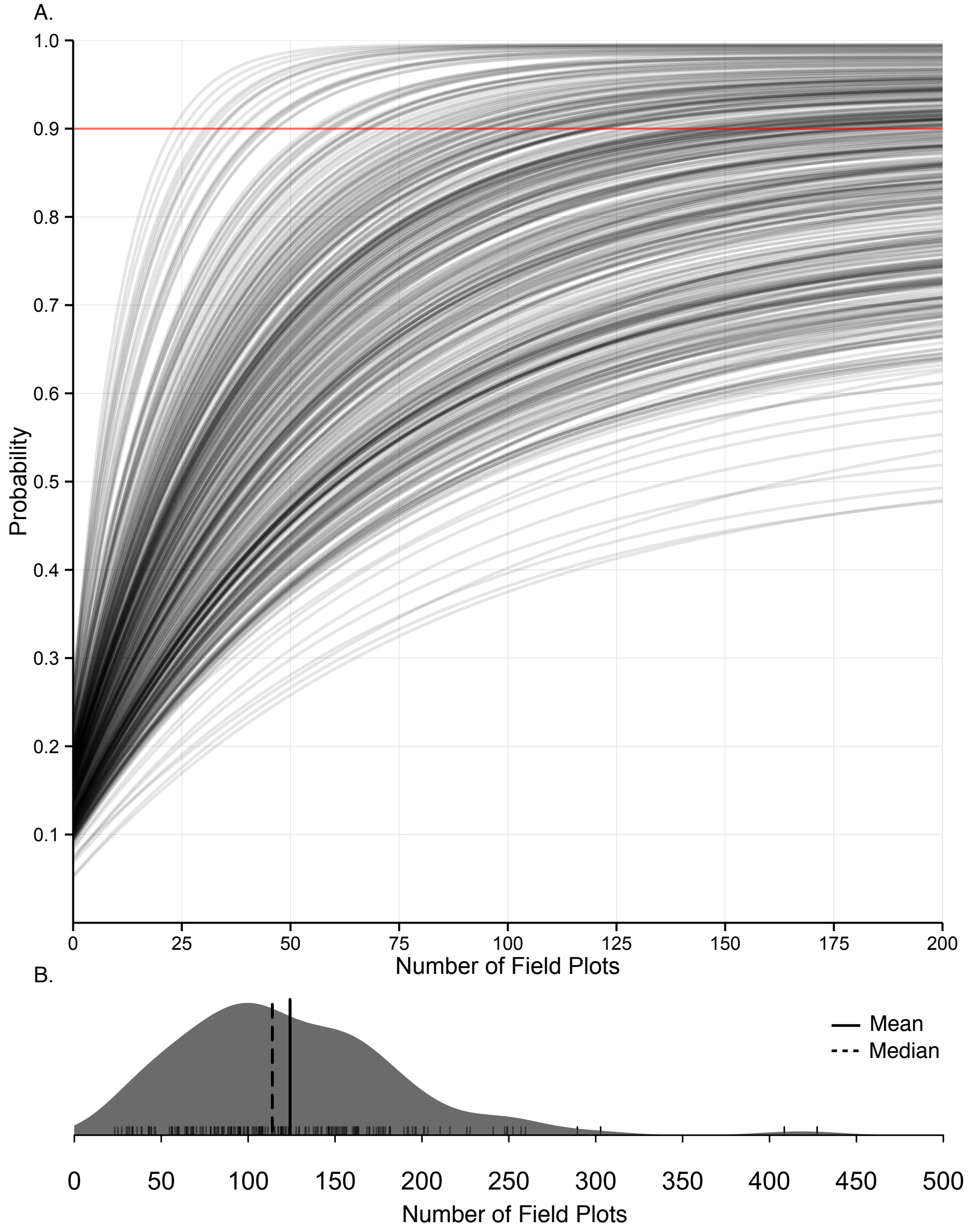

Figure S4. (A) Number of field plots needed to reliably (pr=0.9, red line) estimate the mean model ACD (Mg C ha^{-1}) of a substratum using equal range binning. (B) Frequency distribution of the number of field plots needed to reliably estimate a substratum's mean model ACD.

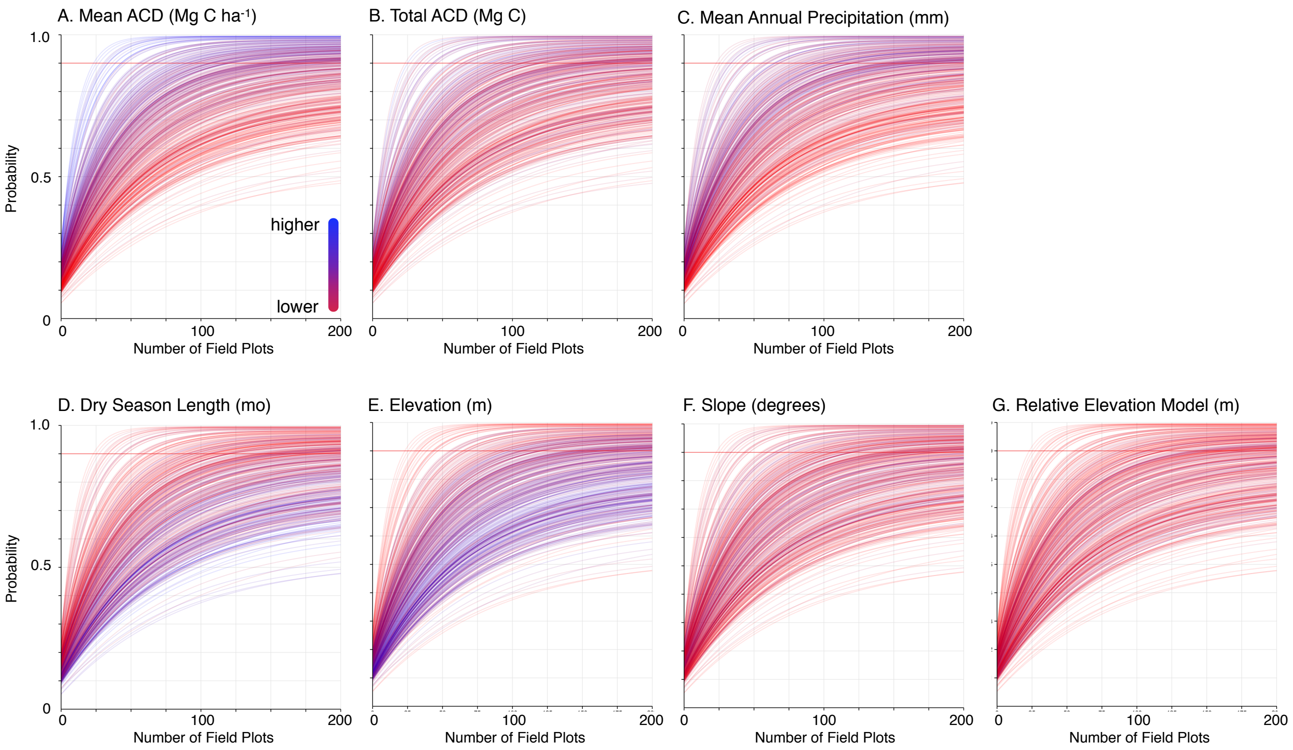

Figure S5. Number of field plots needed to reliably (pr=0.9, red line) estimate the mean model ACD (Mg C ha^{-1}) of a substratum using equal range binning for (A) mean model ACD, (B) total model ACD, (C) mean annual precipitation, (D) dry season length, (E) elevation, (F) slope, and (G) relative elevation. Coloring is same as in Fig S3.
